# Association between healthy sleep patterns and depressive trajectories among college students: a prospective cohort study

**DOI:** 10.1186/s12888-023-04596-0

**Published:** 2023-03-20

**Authors:** Dan Zhang, Yang Qu, Shuang Zhai, Tingting Li, Yang Xie, Shuman Tao, Liwei Zou, Fangbiao Tao, Xiaoyan Wu

**Affiliations:** 1 Department of Maternal, Child and Adolescent Health, School of Public Health, Anhui Medical University, Hefei, China; 2 MOE Key Laboratory of Population Health Across Life Cycle, Hefei, China; 3 NHC Key Laboratory of Study on Abnormal Gametes and Reproductive Tract, Hefei, China; 4 Anhui Provincial Key Laboratory of Population Health and Aristogenics, Anhui Medical University, Hefei, China

**Keywords:** Healthy sleep pattern, Trajectories of depression symptoms, College students

## Abstract

**Background:**

The purpose of this study was to identify different develpment trajectories of depression symptoms during college period, and prospectively investigate the associations healthy sleep patterns with trajectories of depression symptoms among college students from freshman through junior year.

**Methods:**

A total of 999 participants from the College Student Behavior and Health Cohort Study were included between April 2019 and June 2021. Healthy sleep patterns were defined by chronotype, sleep duration, insomnia, snoring, and daytime sleepiness. Latent growth curve model was used to identify trajectories of depression symptoms. Then binary logistic regression was used to examine association of the healthy sleep patterns with these trajectories.

**Results:**

In baseline survey, we found that a total of 100 (10.0%) participants had healthy sleep patterns’ score equal to 5. Then, we used 5 surveys’ data to identify 2 distinct trajectories of depression symptoms during college (decreasing: 82.5%; increasing: 17.5%). The healthy sleep patterns were associated with these trajectories, the better healthy sleep patterns significantly decrease the risk of increasing trajectories of depression symptoms in males (OR: 0.72, 95%CI: 0.54 ~ 0.97, P = 0.031). Moreover, we found out that the healthy sleep patterns of college students can predict the future depressive symptoms in this study (all P < 0.001).

**Conclusion:**

Our findings indicate that the better healthy sleep patterns may significantly decrease the risk of increasing trajectory of depression symptoms only in male college students. The results speak to a need for college student with depression symptoms to identify and address sleep problems when present, which could prevent or reduce depression detriments in later life.

**Supplementary Information:**

The online version contains supplementary material available at 10.1186/s12888-023-04596-0.

## Introduction

Depression affects about 300 million people worldwide and has become a global public health burden [1]. As a distinct period of development straddling the adolescent and young adulthood life stages, the college years are a peak period for onset of many common mental health problems such as depression are especially prominent [2]. In the United States, college students are at high risk for psychological problems [3]. Similarly, over 20% of Chinese college students suffering from depression, and this rate has been growing over the past decade [4, 5]. Thus, the college period is an essential window to identify modifiable of risk factors and to intervene to prevent depression later in life [6], we should gain a better understanding of the nature and change of depression during this period. Noteworthy, previous evidence suggested that depression needs to be considered along a continuum, as prolonged or recurrent episodes of depression are likely to have long-term physical and psychological effects [7, 8], and these findings highlight the importance of examining the effect of long-term patterns about depression. Therefore, it is necessary to reveal the variability and patterns in the development and/or persistence of depression by identifying different depressive trajectories during college.

Sleep problems, the most common precursor of depression, are common throughout the college period [9]. To the best of our knowledge, the multiple sleep behaviors have been proved to be closely related to the depression of college students [10–14], but most studies of their association have typically only examined one dimension of sleep, such as sleep duration or sleep quality. It is worth noting that a variety of sleep behaviors are intricately linked. Therefore, it is important to assess the overall sleep patterns that combine these different sleep behaviors, which may be more predictive of depression changes throughout the college period [10]. Healthy sleep patterns [11], involving multiple sleep behavior including snoring, sleep duration, insomnia, chronotype, and daytime sleepiness, which can reliably reflect the real sleep status of individuals. In previous studies, healthy sleep patterns have been used primarily as a predictor of cardiovascular health and rarely as a predictor of mental health, especially among college students [10, 11]. In addition, gender is often included as a potential covariate in sleep research, but we note that there is no consensus regarding how gender modifies the association between sleep behaviors and depression. Thus, evidence of high quality on how such healthy sleep patterns increase the risk of depression outcomes among college students and whether there are gender differences is lacking.

To address these key evidence gaps, we measured multiple sleep behavior and depressive symptoms for 2 years in a prospective follow-up study among college students. Our repeated survey design also allowed us to examine how changes in depression over time and how healthy sleep patterns predict the trajectories of depression. Consequently, we formulated 3 hypotheses: (1) There was a negative association between healthy sleep patterns and depressive symptoms; (2) the poorer healthy sleep patterns may significantly increase the risk of increasing trajectories of depression symptoms; (3) and there may be gender differences in their association.

## Material and method

### Participants and procedure

Participants were enrolled in the College Student Behavior and Health Cohort Study, a cohort study designed to follow the health-related behaviors, physical and mental health of college students who were in freshman through junior year between April 2019 and June 2021. The cluster of random samples was employed, with the college as the primary sampling unit. Participants were chosen between April and May 2019 from a medical university situated in Hefei, Anhui Province, and a comprehensive university located in Shangrao, Jiangxi Province. At baseline survey (T1), all participants aged 18.82 ± 1.17 years were requested to fill out an electronic questionnaire. We collected 1135 validated questionnaires with baseline information on sociodemographics, health-related behaviors, and mental healthy, with a response rate of 98.6%. Follow-up was conducted every 6 months and surveyed using a questionnaire similar to the baseline. The last round of survey (T5) was conducted with the participants T1 2 year later, between April and June 2021. Finally, a total of 999 valid respondents both have 5 survey data were analyzed.

The study protocol was approved by the Ethics Committee of Anhui Medical University (No. 20,170,291). In accordance with the principles of the Declaration of Helsinki, written informed consent was obtained from all participants prior to the completion of the survey. And upon completion of the survey, a free physical examination report was provided to all study participants.

### Healthy sleep patterns

We used an electronic questionnaire to assess sleep behaviors in freshman at T1, and then an index score of healthy sleep patterns were established, which includes 5 aspects of sleep behavior: chronotype, sleep duration, insomnia, snoring, and excessive daytime sleepiness [10, 11].

*Chronotype*: Chronotype was evaluated by this question (“What chronotype do you think you are: 1) definitely a ‘morning’ person; 2) more a ‘morning’ person than ‘evening’ person; 3) more an ‘evening’ person than a ‘morning’ person; 4) definitely an ‘evening’ person.”) from Morning and Evening Questionnaire-5 (MEQ-5) [12].

#### Sleep duration

Sleep duration was reported as the number of hours slept during the day (including naps).

*Snoring*: Snoring were assessed by a question (“Do you snore or cough middle of night?: 1)No; 2)Yes.”) in the Pittsburgh Sleep Quality Index (PSQI) [13].

#### Excessive daytime sleepiness

Excessive daytime sleepiness were also assessed by PSQI, which is a component of PSQI and scored on a scale of 0 to 3. The higher the score, the more severe the excessive daytime sleepiness.

#### Insomnia

Insomnia symptoms were assessed by the Insomnia Severity Index (ISI) [14]. The ISI is a self-report measurement aimed at assessing the severity of insomnia over the past two weeks. The measure consists of 7 items and total scores range from 0 to 28. The higher the score, the higher the perceived severity of insomnia, with a score of ≥ 9 defined as insomnia and < 9 defined as no insomnia.

Healthy sleep patterns were defined as early chronotype (“morningness” or “morningness than eveningness”); normal sleep duration (7–8 h/day); no insomnia; no snoring; and no excessive daytime sleepiness (“never/rarely” or ”sometimes”). Each sleep behavior was coded 1 if fitting the healthy criterion and 0 if not (See Table S1 for details, available online). The healthy sleep patterns’ score was obtained by summing up the 5 individual sleep behaviors. A higher score indicated a healthier sleep pattern.

### Depression symptoms

The Patient Health Questionnaire 9 (PHQ-9) was used to measure self-reported depressive symptoms in 5 surveys (T1-T5) [15] when participants were in freshman through junior year. The PHQ-9 is a 9-item questionnaire that measures the presence of depressive symptoms in the past month, assessing 9 depressive symptoms according to the DSM-IV, with each item rated on a Likert scale from 0 to 3. The scale scores ranged from 0 to 27, and the higher the score, the more severe the depressive symptoms. As depressive symptoms exist along a continuum, we used the continuous depression score as the primary outcome of our analysis.

### Covariates

Health-related behaviors included cigarette consumption, alcohol consumption, mobile phone addiction, and physical activity. In the Young Risk Behavior Surveillance System questionnaire [16] the two questions were modified to measure current cigarette and alcohol consumption. Cigarette consumption was assessed by “In the past month, how many days have you smoked” Alcohol consumption was assessed by “In the past month, how many days have you drunk”. The answers are recoded into “yes” or “no”. The Self-Assessment Questionnaire for Adolescents with Problematic Mobile Phone Use (SQAPMPU) [17] is a standardized questionnaire developed by Tao et al. to assess adolescent PMPU. The scores ≥ 27 are defined as having mobile phone addition. Physical activity was assessed using International Physical Activity Questionnaire Short Form (IPAQ-SF) [18], IPAQ-SF is one of the most widely used questionnaires to measure the physical activity level of individuals aged 15–59 years, which level was divided into 3 categories: low, moderate and high.

### Data analysis

All statistical tests were two-sided and significance was set at *P* < 0.05. All data were analyzed in R version 4.1.0, and the graphs were plotted using Graphpad Prism 8. In present study, continuous variables of the normal distribution were represented as mean and standard deviation (SD), while categorical variables were summarized using frequencies (n) and percentages (%).

First, we used pearson correlation to investigate the cross-sectional and longitudinal associations between healthy sleep patterns (T1) and depression of college students in 5 surveys (T1-T5). Second, we conducted latent growth curve model (LGCM) to identify latent trajectories of depression symptoms using 5 surveys’ data of the PHQ-9. LCGM assumes that individuals have the same slope and intercept within the same subgroup, and that there are no individual differences in development trajectories within the category group, thus dividing the participants with heterogeneity into several subgroups, the developmental trajectories of each subgroup and the differences of individual development were described [19]. Briefly, LGCM stratifies individuals from a population into multiple latent classes. The optimal model of development trajectory is determined according to the following principles: (1) the smaller the absolute value of the Bayesian information criterion (BIC) is, the closer the model is to 0, indicating the model fitting; (2) the Entrop values of each trajectory grouping reflect the fitting of the model. When Entrop value > 0.70, it indicates a good fit; (3) the results of LMR-LRT (Lo-Mendell-Rubin) likelihood ratio test and BLRT (Bootstrap)-based likelihood ratio test were significant (*P* < 0.05) ; (4) the proportion of each subgroup was not less than 3%. In this study, only 2 categories met all the criteria, so we finally chosen 2-class solution as the best solution.

Then, Chi-square tests were used to assess the demographic characteristics stratified by trajectories of depression symptoms. Last, the associations between healthy sleep patterns (T1) and trajectories of depression symptoms were assessed with binary logistic regression, and adjusted for confounding factors including household economic status, only child, parental depression history, smoking consumption, alcohol consumption, mobile phone addiction and physical activity. Existing findings showed that increasing trajectories of depression symptoms was characterized by worse mental health, worse recovery, more disabilityand more behavioral problems compared to decreasing trajectories of depression symptoms. Therefore, the healthier group, decreasing trajectories of depression symptoms was recognized as the control group.

## Results

### Participant characteristics according to healthy sleep patterns’ score

Baseline characteristics of the 999 study participants according to the index score of the healthy sleep patterns are presented in Table 1. A total of 100 (10.0%) participants had healthy sleep patterns’ score equal to 5. The mean level of chronotype, sleep duration, insomnia, snoring, excessive daytime sleepiness and healthy sleep patterns were 0.43 ± 0.49, 0.38 ± 0.49, 0.86 ± 0.35, 0.91 ± 0.29, 0.72 ± 0.45 and 3.33 ± 0.96, respectively. Males, non-parental depression history, non-smoking, non-drinking, mobile phone addiction and low physical activity college students are more likely to have a healthier sleep pattern.

**Table 1:** Baseline characteristics of participants according to healthy sleep score (N = 999)

Baseline characteristics	Healthy sleep pattern				
	0–1	2	3	4	5
**Gender**					
Males	15 (4.0)	56 (14.9)	133 (35.3)	134 (35.5)	39 (10.3)
Females	16 (2.6)	80 (12.9)	267 (42.9)	198 (31.8)	61 (9.8)
**Registered residence**					
Rural	27 (3.2)	119 (13.9)	332 (38.9)	284 (33.3)	92 (10.8)
Urban	4 (2.8)	17 (11.7)	68 (46.9)	48 (33.1)	8 (5.5)
**Only child**					
Yes	10 (4.4)	30 (13.0)	103 (44.6)	75 (32.5)	13 (5.6)
No	21 (2.8)	106 (13.8)	297 (38.7)	257 (33.5)	87 (11.3)
**Father’s education**					
< 12 years	26 (2.9)	121 (13.4)	363 (40.1)	301 (33.2)	95 (10.5)
≥ 12 years	5 (5.4)	15 (16.1)	37 (39.8)	31 (33.3)	5 (5.4)
**Maternal education**					
< 12 years	29 (3.0)	127 (13.3)	384 (40.3)	315 (33.1)	98 (10.3)
≥ 12 years	2 (4.4)	9 (19.6)	16 (34.8)	17 (37.0)	2 (4.3)
**Parental depression history**					
No	25 (2.6)	129 (13.5)	385 (40.3)	319 (33.4)	97 (10.2)
Yes	6 (13.6)	7 (15.9)	15 (34.1)	13 (29.5)	3 (6.8)
**Household economic status**					
Low	6 (2.4)	45 (18.4)	95 (38.8)	76 (31.0)	23 (9.4)
Moderate	24 (3.3)	86 (12.2)	282 (39.9)	241 (34.1)	74 (10.5)
High	1 (2.1)	5 (10.6)	23 (48.9)	15 (31.9)	3 (6.4)
**Smoking consumption**					
No	21 (2.3)	114 (12.6)	373 (41.4)	300 (33.3)	94 (10.4)
Yes	6 (10.9)	14 (25.5)	22 (40.0)	12 (21.8)	1 (1.8)
**Alcohol consumption**					
No	19 (2.5)	95 (12.4)	316 (41.4)	251 (32.9)	83 (10.9)
Yes	13 (5.1)	41 (17.4)	84 (35.7)	81 (34.5)	17 (7.2)
**Mobile phone addiction**					
No	7 (0.9)	77 (10.2)	306 (40.5)	275 (36.4)	91 (12.0)
Yes	24 (9.9)	59 (24.3)	94 (38.7)	57 (23.5)	9 (3.7)
**Physical activity**					
Low	12 (8.9)	21 (15.7)	51 (38.1)	43 (32.1)	7 (5.2)
Moderate	13 (2.8)	60 (13.0)	203 (43.8)	145 (31.3)	42 (9.1)
High	6 (1.5)	55 (13.7)	146 (36.3)	144 (35.8)	51 (12.7)

Note: The figures in () are composition ratio or detection rate/%

### Association between healthy sleep patterns (T1) and depression (T1-T5)

**Fig. 1:**
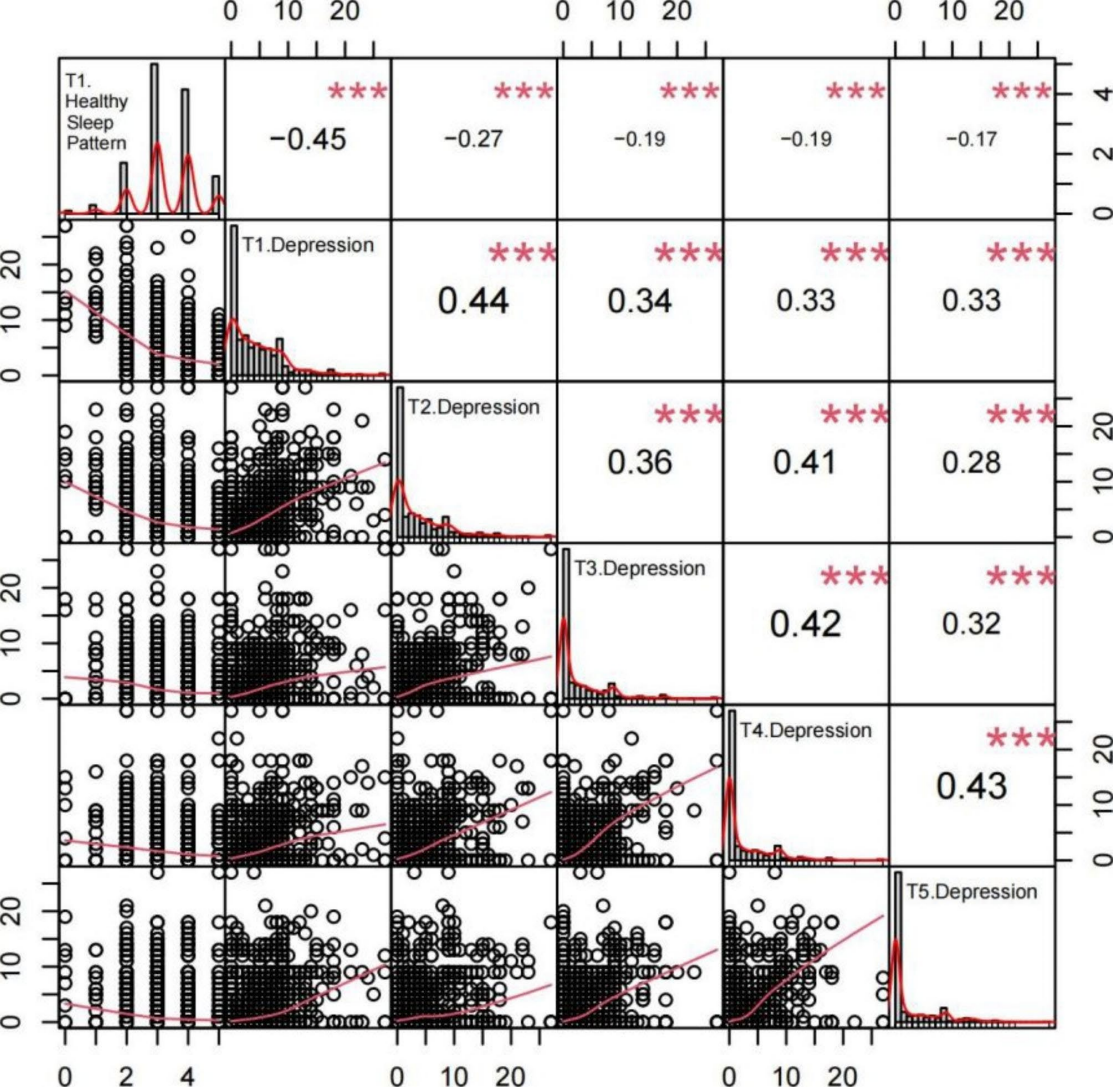
The association between healthy sleep patterns and depression Note: ***: *P*<0.001

Figure 1 shows the association between sleep patterns (T1) and depression (T1-T5). Consistent with our hypothesis, the results suggested that at baseline(T1) the better healthy sleep patterns’ scores, the lower the depression scores (*r*=-0.45, *P* < 0.001). Moreover, healthy sleep patterns at baseline (T1) was negatively correlated (all *t* < 0) with depression score at follow-up (T2-T4) (all *P* < 0.001), and the correlation decreases with time (See Table S2 for details, available online).

### Trajectories of depression symptoms

**Fig. 2:**
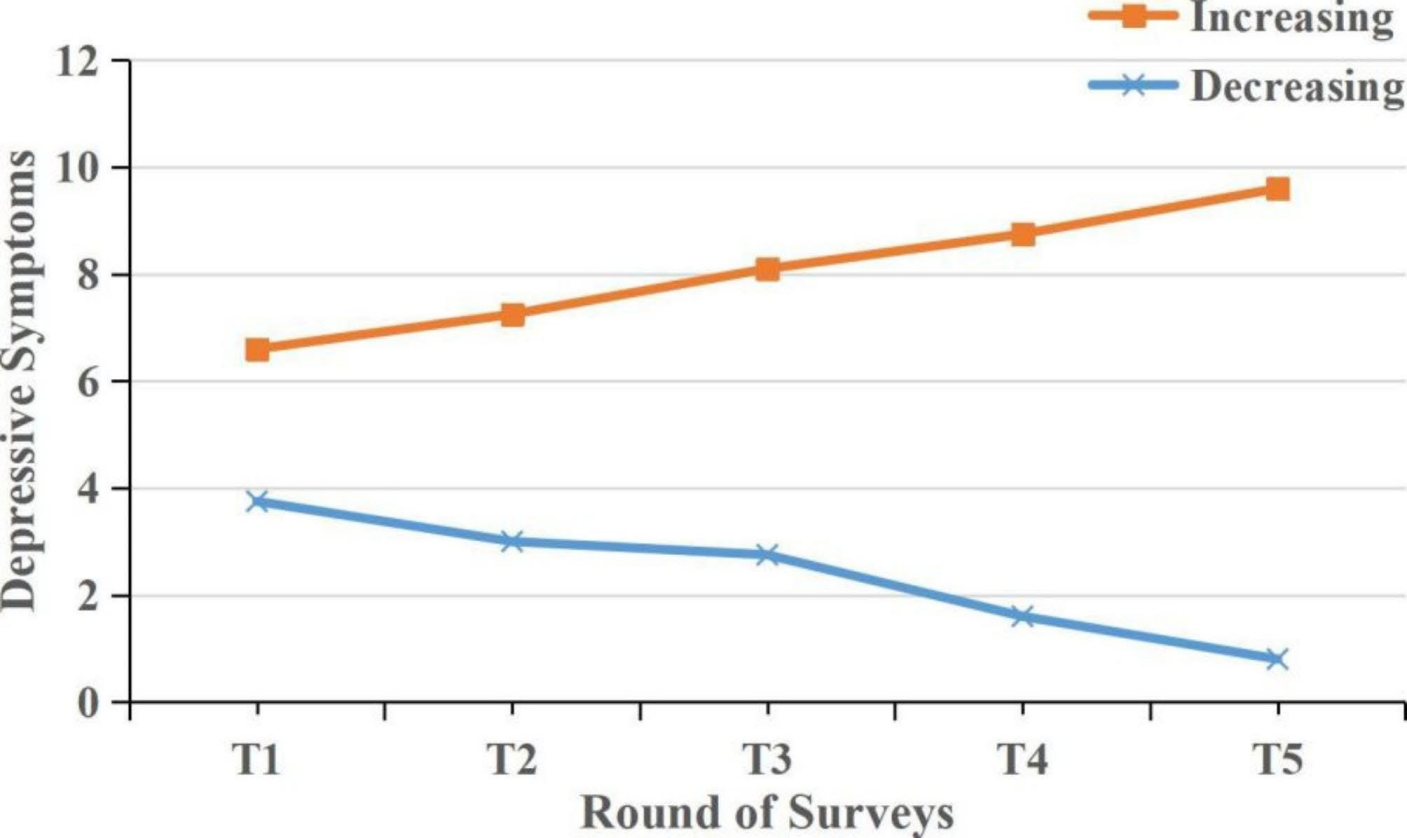
The trajectories of depression symptoms

Among the sample of 999 college students, 2 heterogeneous trajectories of depression symptoms were derived (See Table S3 for details, available online). First, the decreasing trajectory (824 college students [82.5%]) included individuals who had consistently decrease levels of depression symptoms. Second, the increasing trajectory (175 individuals [17.5%]) included individuals with consistently increase levels of depression symptoms (Fig. 2).

### Association of healthy sleep patterns with trajectories of depression symptoms

**Fig. 3:**
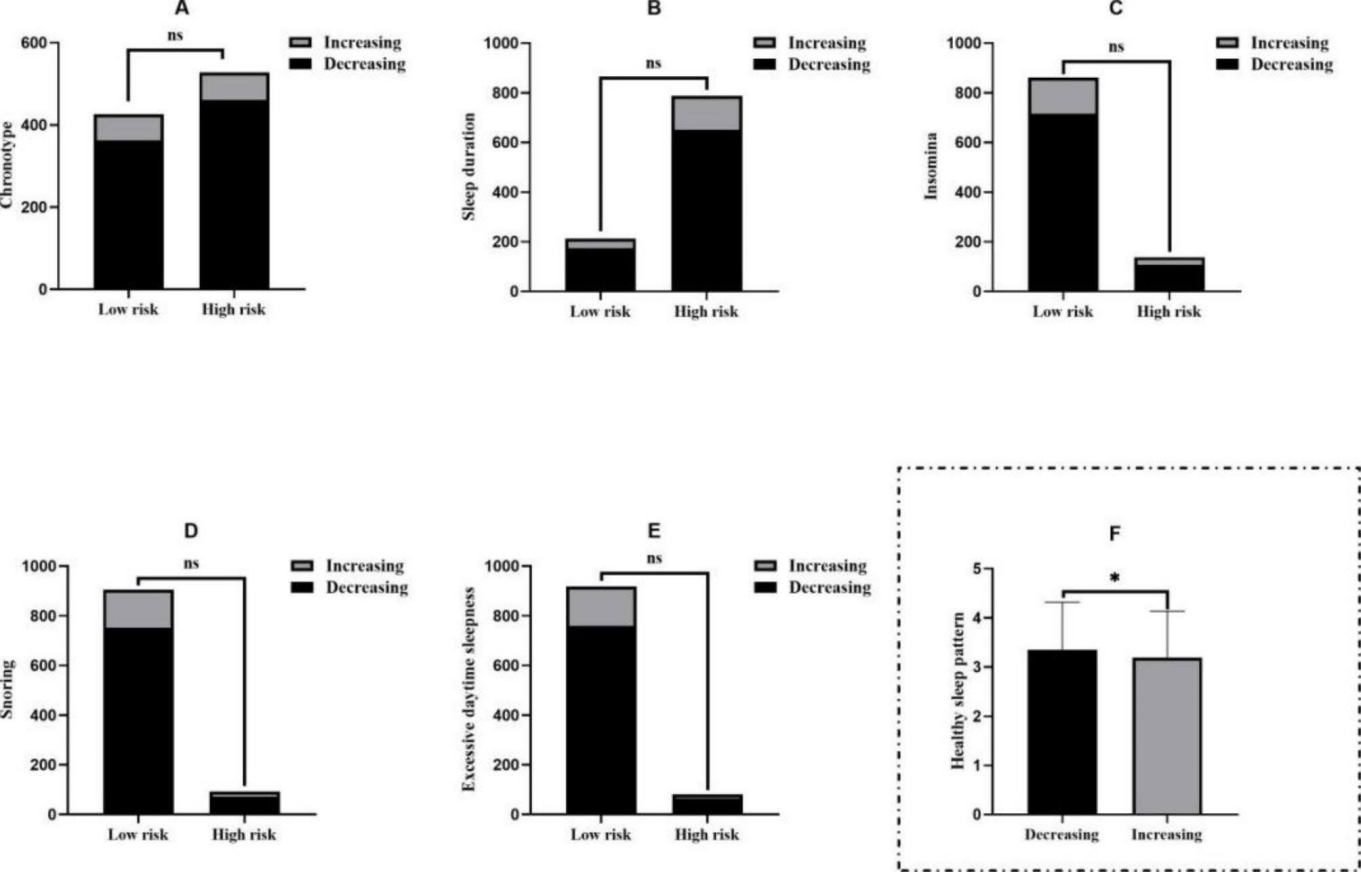
The association between individual sleep behaviors, healthy sleep patterns and different trajectories of depression symptoms Note: ns: no significant; **: P<0.001

There were statistical differences in the levels of healthy sleep patterns in this study among different trajectories of depression symptoms (3.36 ± 0.96, 3.19 ± 0.95, *t* = 2.087, *P* = 0.037; Fig. 3, Table S4). Table 2 describes the results of individual sleep behavior, healthy sleep patterns and different trajectories of depression symptoms analysis using the binary logistic regression. The results indicated that only chronotype (*OR*: 0.71, 95%*CI*: 0.50 ~ 1.00, *P* = 0.048) and healthy sleep patterns (*OR*: 0.84, 95%*CI*: 0.71 ~ 0.99, *P* = 0.038) were statistically negatively correlated with trajectories of depression symptoms. After adjusting for variables such as household economic status, only child, smoking consumption, alcohol consumption, parental depression history, mobile phone addiction and physical activity, the negative correlation disappears (Table 2). Then, we performed gender-stratified analysis to examine the association between healthy sleep patterns and different trajectories of depression symptoms (See Figure S1 for details, available online). In the adjusted model, the effect of healthy sleep patterns on trajectories of depression symptoms was kept statistically significant only in male, the level of healthy sleep patterns were lower in increasing trajectories group (*OR*: 0.72, 95%*CI*: 0.54 ~ 0.97, *P* = 0.031). Results from the sensitivity analyses are shown in Table S5 (available online), we added the variables baseline anxiety and baseline depression into the fully adjusted models, and majority of the results remained consistent as above.

**Table 2:** Logistic regression of healthy sleep pattern and different trajectories of depression symptoms

Variables	Crude MODEL			Adjusted MODEL ^a^	
	*OR* (95%*CI*)	*P* value		*OR* (95%*CI*)	*P* value
**Individual component**					
Chronotype^#^	0.71 (0.50 ~ 1.00)	0.048		0.71 (0.50 ~ 1.02)	0.061
Sleep duration^#^	1.10 (0.74 ~ 1.64)	0.634		1.17 (0.78 ~ 1.76)	0.449
Insomnia^#^	0.76 (0.48 ~ 1.20)	0.246		0.77 (0.48 ~ 1.24)	0.284
Snoring^#^	0.66 (0.39 ~ 1.13)	0.131		0.66 (0.37 ~ 1.17)	0.151
Excessive daytime sleepiness^#^	0.81 (0.45 ~ 1.44)	0.471		0.83 (0.46 ~ 1.52)	0.549
**Total**					
Healthy sleep pattern	0.84 (0.71 ~ 0.99)	0.038		0.85 (0.71 ~ 1.03)	0.093
**Male**					
Healthy sleep pattern	0.70 (0.54 ~ 0.91)	0.008		0.72 (0.54 ~ 0.97)	0.031
**Female**					
Healthy sleep pattern	0.98 (0.78 ~ 1.23)	0.873		0.97 (0.76 ~ 1.24)	0.830

**Note**: ^#^: low risk was recognized as the control group. control group

Adjusted MODEL controlled household economic status, only child, parental depression history, smoking consumption, alcohol consumption, mobile phone addiction and physical activity

## Discussion

To the best of our knowledge, this study is the first prospective study examining associations between healthy sleep patterns and trajectories of depression symptoms in college students. First, we found out that the healthy sleep patterns of college students can predict the future depressive symptoms in this study. Then, we identified 2 distinct trajectories (decresing: 82.5%; incresing: 17.5%) of depression symptoms during college. And healthy sleep patterns were associated with these trajectories, the better healthy sleep patterns significantly decrease the risk of increasing trajectories of depression symptoms in male college students. These findings suggest that examining healthy sleep patterns could help identify groups with severe and chronic depression symptoms (i.e., in the college-persistent trajectory) who should be prioritized for early intervention.

The healthy sleep patterns used by this study (early chronotype, sleep 7–8 h per day, never or rarely insomnia, no snoring, and no frequent excessive daytime sleepiness) provides a better frame of reference for sleep and is also of value in identifying high-risk individuals. Currently, healthy sleep patterns are widely used in middle-aged and older adults, while no studies of young adults are available. In present study, we found that a total of 100 (10.0%) participants had a healthy sleep patterns’ score equal to 5, which was lower than in previous studies of middle-aged and the elderly adults from UK Biobank (21.4%) [11]. This may be due to the different lifestyles of younger and middle-aged and older adults, with college students tending to sleep later, for shorter sleep duration and so on [20], thus exhibiting lower healthy sleep patterns. And the lower healthy sleep patterns indicate that college students may have higher health risks.

Based on developmental theory suggesting that most individuals experience continuously lower levels of psychological distress during the transition from adolescence to young adulthood [21], which is also consistent with our study. Our data suggest that an estimated 82.5% of college students were classified in the consistently decrease levels of depression symptoms, and only 17.5% of college students were classified in the consistently increase levels of depression symptoms. We found only 2 depressive trajectories in the college students, which are different from Kwong ASF et al. [7] and Weavers B et al. [22], who found 5 classes across the age span of 10–24 years and 4 classes between ages 10.5 and 25 years, respectively. So far, we only found that in a longitudinal study from childhood to adolescence, Prinzie P et al. [23] identified 2 depression trajectories(increasing: 5.9%). One possible explanation for this difference is the age range is inconsistent. Most previous studies have focused on changes in the trajectory of adolescent depression over a larger time span, however, our study of college students from freshman to junior only has a shorter time span.

Although sleep problems are better predictors of depression than other symptom, there are no previous studies focusing on the effects of healthy sleep patterns on mental health. In present study, healthy sleep patterns predict future depressive symptoms, and the lower the healthy sleep patterns, the worse the future depressive symptoms. Our findings are supported by several previous studies assessing the relations of individual sleep behavior with depressive symptoms, in which later chronotype [24], short or prolonged sleep duration [25, 26], insomnia [3], snoring [27], and excessive daytime sleepiness [28] were found to be associated with increased risks of depressive symptoms among college students. Meanwhile, our results also highlight that low healthy sleep patterns during college is associated with both short-term and long-term mental healthy consequences. We found that chronotype and healthy sleep patterns were positively correlated with trajectories of depression symptoms. However, after adjusting for variables, we found no association between individual sleep behaviors, healthy sleep patterns and depressive trajectories, which is inconsistent with our hypothesis. Given that gender-specific college students show distinct patterns of socio-demographic characteristics, sleep behaviors, and mental healthy, it is likely that these differential characteristics may influence the association between healthy sleep patterns and depressive trajectories. Therefore, we furtherly explore the gender difference between the healthy sleep patterns and depressive trajectories.

Interestingly, the distribution of healthy sleep patterns (score equal to 5: 10.3% vs. 9.8%) and depressive trajectories (increasing depression: 17.8% vs. 17.4%) was uniform among college students of different gender, however, after performed gender-stratified, we found that the poorer healthy sleep patterns may significantly increase the risk of increased depression in males. Our results are different from Supartini A et al. [29] and Foster SN et al. [30], who found that late bedtime, short sleep duration and insomnia were independently associated with depressive symptoms in females. However, these foregoing studies focused on the association between sleep and depressive symptoms, not the depressive trajectory. Chang LY et al. [31] has given a more reasonable explanation that potential gender differences in the association being examined may be due to the differences in sleep characteristics, compared to females, males generally have more sleep problems, and these gender differences may affect how sleep behaviors impacts the depression differently for males and for females. Additionally, the higher propensity to seek support when encountering sleep problems in females than in males and thereby get better treatment may help decrease the negative impact of sleep problems on depression in females [31]. Therefore, based on the causal relationship between sleep patterns and depressive trajectories in male college students, we can consider future measures against male college students’ adverse sleep behaviors to reduce the risk of increased future depression.

Several potential mechanisms could explain the observed associations between the healthy sleep patterns and the reduced risk of depression [32]. First, a compelling body of evidence demonstrates that brain activities involving the regulation of mood behavior are comprehensively influenced by the circadian activity, and sleep as an individual’s circadian rhythm, increases in depressed mood are usually associated with the abnormal sleep behavior. Second, the melatonin-related circadian hypothesis and the HPA axis dysregulation hypothesis of depression are closely related because melatonin and cortisol are interacting hormones, and when secretions which regulate circadian rhythms are blunted, those which elevate mood are similarly affected, thus producing a depressed emotional state. Last, normal sleep is essential for regulating the amygdala response to various emotional stimuli, disturbances in sleep may initiate the appearance of depression disorders.

This study has limitations. Firstly, this study used self-report to evaluate the healthy sleep patterns and depressive symptoms, so it may not be able to avoid reporting bias; however, the large sample size available for analysis and using repeated assessments reduce the likelihood of this having a meaningful impact on the results presented here. Secondly, although we used 5 survey data to fit depression trajectories, the time interval was short and didn’t reflect trends in depression dynamics throughout the college years, which may be why our analyses support a two-class model. Thirdly, because of the presence of sleep items in PHQ9, the association we observed between sleep patterns and depressive trajectories may have been influenced by sleep items, but when we later deleted the sleep items, the trajectories were re-fitted; No significant change was found in the results. In addition, the PHQ score was not sufficient to diagnose depression, and because of the lack of objective assessment, the results we observed were only about the association between sleep patterns and depressive symptom scores. At last, the study objects were all college students, which have their own group characteristics, so the study may have selection bias, the results to the general population extrapolation may be limited.

Nonetheless, the current study has several strengths. First, this study is the first prospective study examining associations between healthy sleep patterns and trajectories of depression symptoms in college students. Second, the healthy sleep patterns used by this study combining various sleep behaviors and provides a better frame of reference for sleep. Additionally, most previous studies of the association between sleep and depression have focused on cross-sectional studies, and our repeated measures design also allowed us to examine the predictive effect of healthy sleep patterns on depressive symptoms in college students at different stages, further addressed this gap. Last, examinations of the links between healthy sleep patterns and subsequent depressive trajectories helped to enhance the current understanding of risk factors of developmental patterns of depressive trajectories.

## Conclusion

In summary, we found that college students’ healthy sleep patterns were related to future depressive symptoms. Furthermore, the better healthy sleep patterns may significantly decrease the risk of increasing trajectories of depression symptoms only in male college students. Our findings add to basic etiologic research regarding developmental pathways of depression during college as well as the prospective association of sleep behaviors with the probability of depressive trajectory group membership. This speaks to a need for college student to identify and address sleep problems when present especially in males, which could prevent or reduce depression detriments in later life.

## Electronic supplementary material

Below is the link to the electronic supplementary material.

Supplementary Material 1: Gender differences in the association between different depressive trajectories and healthy sleep pattern

Supplementary Material 2: Related supplementary tables

## Data Availability

The datasets generated and analysed during the current study are not publicly available because the author does not have permission to share the data but are available from the corresponding author on reasonable request.
