# Supplementary material for: Association between healthy sleep patterns and depressive trajectories among college students: a prospective cohort study
Source: BMC Psychiatry. 2023 Mar 20;23:182. doi: 10.1186/s12888-023-04596-0 (PMC10026494; doi:10.1186/s12888-023-04596-0)
Supplement: Supplementary file 1 — Supplementary Material 1: Gender differences in the association between different depressive trajectories and healthy sleep pattern [file 12888_2023_4596_MOESM1_ESM.docx]

**
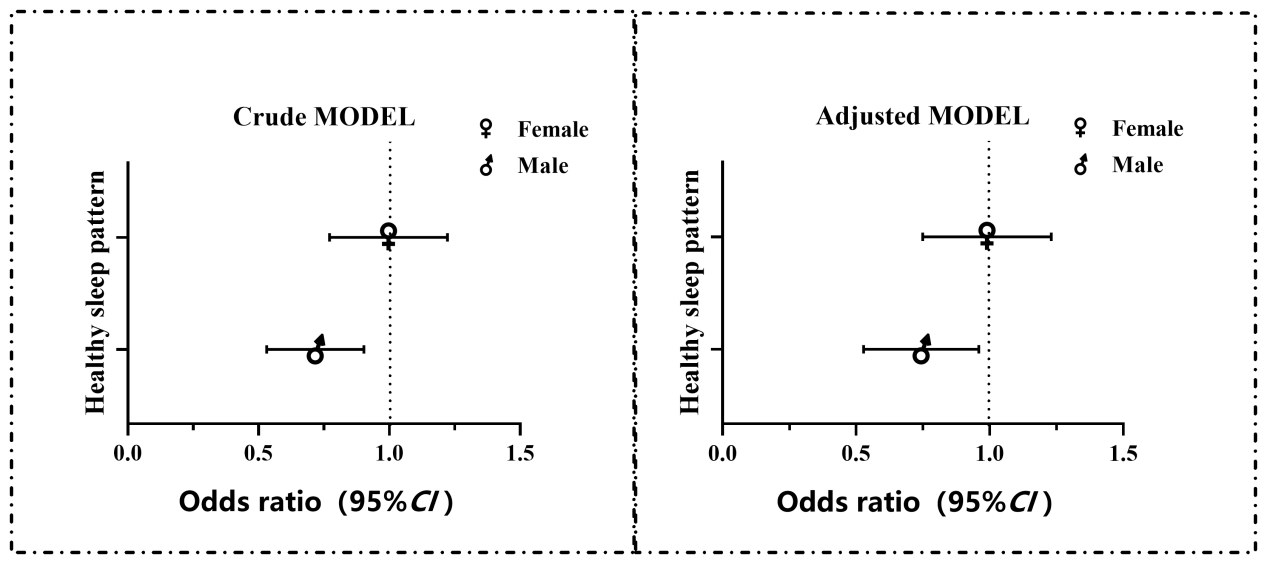
**

**Fig.S1** Gender differences in the association between different depressive trajectories and healthy sleep pattern

**Note:** Adjusted MODEL control household economic status, only child, parental depression history, smoking consumption, alcohol consumption, mobile phone addiction and physical activity.
