# Supplementary material for: Association between healthy sleep patterns and depressive trajectories among college students: a prospective cohort study
Source: BMC Psychiatry. 2023 Mar 20;23:182. doi: 10.1186/s12888-023-04596-0 (PMC10026494; doi:10.1186/s12888-023-04596-0)
Supplement: Supplementary file 2 — Supplementary Material 2: Related supplementary tables [file 12888_2023_4596_MOESM2_ESM.docx]

**Table S1**

Development of healthy sleep pattern scores for Chinese college students

| **Risk Indexes** | **Score** | **Risk level** | |
| --- | --- | --- | --- |
|  |  | **Low** | **High** |
| **Chronotype** |  | 1 | 0 |
| Morningness | 1 |  |  |
| Morningness than eveningness | 1 |  |  |
| Eveningness than morningness | 0 |  |  |
| Eveningness | 0 |  |  |
| **Sleep duration** |  | 1 | 0 |
| Normal (7–8 h) | 1 |  |  |
| Short (<7 h) | 0 |  |  |
| Long (>8 h) | 0 |  |  |
| **Insomnia** |  | 1 | 0 |
| No | 1 |  |  |
| Yes | 0 |  |  |
| **Snoring** |  | 1 | 0 |
| No | 1 |  |  |
| Yes | 0 |  |  |
| **Excessive daytime sleepiness** |  | 1 | 0 |
| Never/rarely(0) | 1 |  |  |
| Sometimes(1) | 1 |  |  |
| Often(2) | 0 |  |  |
| All of the time(3) | 0 |  |  |
| **Healthy sleep pattern** | 0~5 |  |  |

**Table S2.**

The association between healthy sleep patterns and depression

| **Continuous Variable** | **Healthy**  **Sleep pattern** | **T1**  **Depression** | **T2**  **Depression** | **T3 Depression** | **T4**  **Depression** | **T5**  **Depression** |
| --- | --- | --- | --- | --- | --- | --- |
| Healthy sleep pattern | 1.00 |  |  |  |  |  |
| T1 Depression | -0.45*** | 1.00 |  |  |  |  |
| T2 Depression | -0.28*** | 0.44*** | 1.00 |  |  |  |
| T3 Depression | -0.19*** | 0.34*** | 0.36*** | 1.00 |  |  |
| T4 Depression | -0.19*** | 0.33*** | 0.41*** | 0.42*** | 1.00 |  |
| T5 Depression | -0.17*** | 0.33*** | 0.29*** | 0.32*** | 0.43*** | 1.00 |

**Note：**The figures in () are composition ratio or detection rate/%

***：*P*<0.001

**Table S3**

Latent class growth model for different groups of depression (n=999)

| **Class** | **Information Criteria** | | |  | **Loglikelihood ratio test (*P* value)** | | | |
| --- | --- | --- | --- | --- | --- | --- | --- | --- |
|  | **AIC** | **BIC** | **aBIC** |  | **LMR** | **BLRT** | **Entrop** | **Class counts** |
| 1 | 28376.721 | 28425.789 | 28394.028 |  |  |  |  |  |
| 2 | 27968.090 | 28031.878 | 27990.589 |  | **0.0028** | **0.000** | **0.929** | **824/175** |
| 3 | 27731.714 | 27810.222 | 27759.405 |  | 0.3629 | 0.000 | 0.962 | 766/61/172 |
| 4 | 27600.541 | 27693.769 | 27633.424 |  | 0.4012 | 0.000 | 0.954 | 36/129/109/725 |
| 5 | 27557.306 | 27665.254 | 27595.381 |  | 0.6085 | 0.000 | 0.926 | 37/55/60/152/695 |

**Table S4**

Baseline characteristics of participants according to trajectories of depression symptoms (N=999).

| **Baseline characteristics** | **Total sample**  **(N=999)** | **Trajectories of depression symptoms** | | ***χ*^2^/*t*** | ***P* value** |
| --- | --- | --- | --- | --- | --- |
|  |  | **Decreasing (N=824)** | **Increasing (N=175)** |  |  |
| **Gender** |  |  |  | 0.03 | 0.869 |
| Males | 377 (37.7) | 310 (82.2) | 67 (17.8) |  |  |
| Females | 622 (62.3) | 514 (82.6) | 108 (17.4) |  |  |
| **Registered residence** |  |  |  | 1.18 | 0.277 |
| Rural | 854 (85.5) | 709 (83.0) | 145 (17.0) |  |  |
| Urban | 145 (14.5) | 115 (79.3) | 30 (20.7) |  |  |
| **Only child** |  |  |  | 6.12 | 0.013 |
| Yes | 231 (23.1) | 178 (77.1) | 53 (22.9) |  |  |
| No | 768 (76.9) | 646 (84.1) | 122 (15.9) |  |  |
| **Father’s education** |  |  |  | 0.43 | 0.512 |
| <12 years | 906 (90.7) | 745 (82.2) | 161 (17.8) |  |  |
| ≥12 years | 93 (9.3) | 79 (84.9) | 14 (15.1) |  |  |
| **Maternal education** |  |  |  | 0.40 | 0.526 |
| <12 years | 953 (95.4) | 785 (82.4) | 168 (17.6) |  |  |
| ≥12 years | 46 (4.6) | 39 (84.8) | 7 (15.2) |  |  |
| **Parental depression history** |  |  |  | 0.014 | 0.906 |
| No | 955 (95.6) | 788 (82.5) | 167 (17.5) |  |  |
| Yes | 44 (4.4) | 36 (81.8) | 8 (18.2) |  |  |
| **Household economic status** |  |  |  | 8.38 | 0.015 |
| Low | 245 (24.6) | 192 (78.4) | 53 (21.6) |  |  |
| Moderate | 707 (70.8) | 598 (84.6) | 109 (15.4) |  |  |
| High | 47 (4.7) | 34 (72.3) | 13 (27.7) |  |  |
| **Smoking consumption** |  |  |  | 0.08 | 0.780 |
| Yes | 55 (5.5) | 46 (83.6) | 9 (16.4) |  |  |
| No | 902 (90.3) | 741 (82.2) | 161 (17.8) |  |  |
| **Alcohol consumption** |  |  |  | 0.57 | 0.452 |
| Yes | 235 (23.5) | 190 (80.9) | 45 (19.1) |  |  |
| No | 764 (76.5) | 634 (83.0) | 130 (17.0) |  |  |
| **Mobile phone addiction** |  |  |  | 0.223 | 0.637 |
| Yes | 243 (24.3) | 198 (81.5) | 45 (18.5) |  |  |
| No | 756 (75.7) | 626 (82.8) | 130 (17.2) |  |  |
| **Physical activity** |  |  |  | 2.51 | 0.286 |
| Low | 134 (13.4) | 106 (79.1) | 28 (20.9) |  |  |
| Moderate | 463 (46.3) | 378 (81.6) | 85 (18.4) |  |  |
| High | 402 (40.2) | 340 (84.6) | 62 (15.4) |  |  |
| **Chronotype** |  |  |  | 3.826 | 0.050 |
| Low risk | 426 (57.4) | 363 (85.2) | 63 (14.8) |  |  |
| High risk | 426 (42.6) | 461 (80.5) | 67 (19.5) |  |  |
| **Sleep duration** |  |  |  | 0.031 | 0.861 |
| Low risk | 212 (21.2) | 174 (82.1) | 38 (17.9) |  |  |
| High risk | 787 (78.8) | 650 (82.6) | 137 (17.4) |  |  |
| **Insomnia** |  |  |  | 1.98 | 0.160 |
| Low risk | 861 (86.2) | 716 (83.2) | 145 (16.8) |  |  |
| High risk | 138 (13.8) | 108 (78.3) | 30 (21.7) |  |  |
| **Snoring** |  |  |  | 1.82 | 0.177 |
| Low risk | 906 (90.7) | 752 (83.0) | 154 (17.0) |  |  |
| High risk | 93 (9.3) | 72 (77.4) | 21 (22.6) |  |  |
| **Excessive daytime sleepiness** |  |  |  | 0.74 | 0.391 |
| Low risk | 918 (91.9) | 760 (82.8) | 158 (17.2) |  |  |
| High risk | 81 (8.1) | 64 (79.0) | 17 (21.0) |  |  |
| **Age** | 18.8 ± 1.17 | 18.80 ± 1.16 | 18.89 ± 1.24 | -0.882 | 0.784 |
| **BMI** | 20.7 ± 2.61 | 20.74 ± 2.56 | 20.67 ± 2.81 | 0.307 | 0.192 |
| **Healthy sleep pattern** | 3.33 ± 0.96 | 3.36 ± 0.96 | 3.19 ± 0.95 | 2.087 | 0.037 |

**Note：**The figures in () are composition ratio or detection rate/%

**TableS5**

Sensitivity analysis about association of healthy sleep pattern and different trajectories of depression symptoms

| **Variables** | **MODEL 1** | |  | **MODEL 2** | |
| --- | --- | --- | --- | --- | --- |
|  | ***OR* (95%*CI*)** | ***P* value** |  | ***OR* (95%*CI*)** | ***P* value** |
| **Individual component** |  |  |  |  |  |
| Chronotype^#^ | 0.72 (0.51~1.02) | 0.066 |  | 0.72 (0.51~1.03) | 0.072 |
| Sleep duration^#^ | 1.19 (0.79~1.79) | 0.413 |  | 1.18 (0.79~1.78) | 0.423 |
| Insomnia^#^ | 0.71 (0.42~1.20) | 0.199 |  | 0.73 (0.43~1.24) | 0.244 |
| Snoring^#^ | 0.65 (0.36~1.17) | 0.151 |  | 0.67 (0.37~1.21) | 0.181 |
| Excessive daytime sleepiness^#^ | 0.84 (0.45~1.57) | 0.591 |  | 0.85 (0.45~1.59) | 0.606 |
| **Total** |  |  |  |  |  |
| Healthy sleep pattern | 0.84 (0.68~1.02) | 0.080 |  | 0.84 (0.69~1.03) | 0.096 |
| **Male** |  |  |  |  |  |
| Healthy sleep pattern | 0.71 (0.51~0.99) | 0.042 |  | 0.71 (0.50~0.99) | 0.045 |
| **Female** |  |  |  |  |  |
| Healthy sleep pattern | 0.64 (0.73~1.22) | 0.643 |  | 0.86 (0.72~1.04) | 0.113 |

**Note:** ^#^: low risk was recognized as the control group. control group.

MODEL 1: controlled baseline anxiety on the basis of Adjusted MODEL.

MODEL 2: controlled baseline depression on the basis of MODEL 1.
